# Conjoint bicondylar Hoffa fracture in a child: a rare variant treated by minimally invasive approach

**DOI:** 10.1007/s10195-011-0133-3

**Published:** 2011-04-21

**Authors:** Hitesh Lal, Pankaj Bansal, Rahul Khare, Deepak Mittal

**Affiliations:** 1 Department of Orthopaedics, Postgraduate Institute of Medical Education and Research, Dr. Ram Manohar Lohia Hospital, New Delhi, India; 2 Senior Resident, Orthopaedics, Ram Manohar Lohia Hospital, Room No.102, Doctors’ Hostel, New Delhi, 110001 India

**Keywords:** Conjoint, Hoffa, Fracture, Arthroscopic fixation

## Abstract

A case of conjoint Hoffa-type fracture in a child is presented. Hoffa fracture, i.e., coronal slice fracture of the condyles of the femur, is rare in adults and even rarer in the pediatric population. To date, no case of conjoint bicondylar Hoffa fracture has been reported in the literature. The presented case was successfully treated by arthroscopically assisted internal fixation.

## Introduction

Fracture at the distal end of the femur usually occurs in the sagittal plane. Coronal fracture of the femoral condyle, first described by Hoffa in 1904 [1], is an unusual injury in adults and rarer still in children. Hoffa fracture usually affects a single femoral condyle, more commonly the lateral condyle. A bicondylar pattern of this injury is very rare, and to the best of our knowledge, only six such cases have been described in the literature, all of which were adults [2–6]. To the best of our knowledge, coronal slice fracture of the distal end of the femur presenting as a conjoint bicondylar Hoffa variant has never been reported. We report a case of bicondylar Hoffa fracture in a 9-year-old child, which was successfully treated by arthroscopically assisted internal fixation.

## Case report

**Fig. 1:**
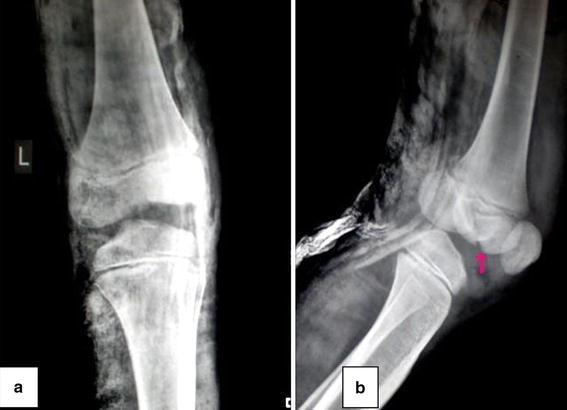
Preoperative anteroposterior **a** and lateral **b** radiographs of the case. The *arrow* in the lateral view indicates the fracture line

**Fig. 2:**
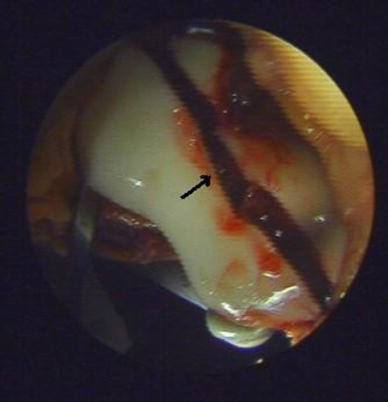
Peroperative arthroscopic panoramic view showing the conjoint bicondylar Hoffa-type fracture. The *arrow* indicates the connecting bridge between the two hemicondyles

**Fig. 3:**
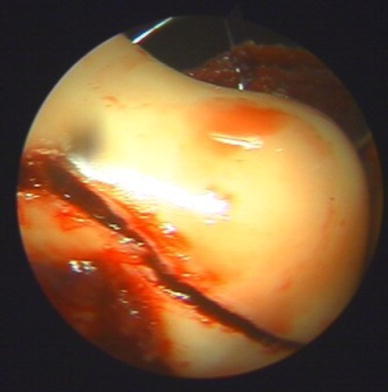
Peroperative arthroscopic panoramic view showing provisional reduction of the fracture using two guide wires

**Fig. 4:**
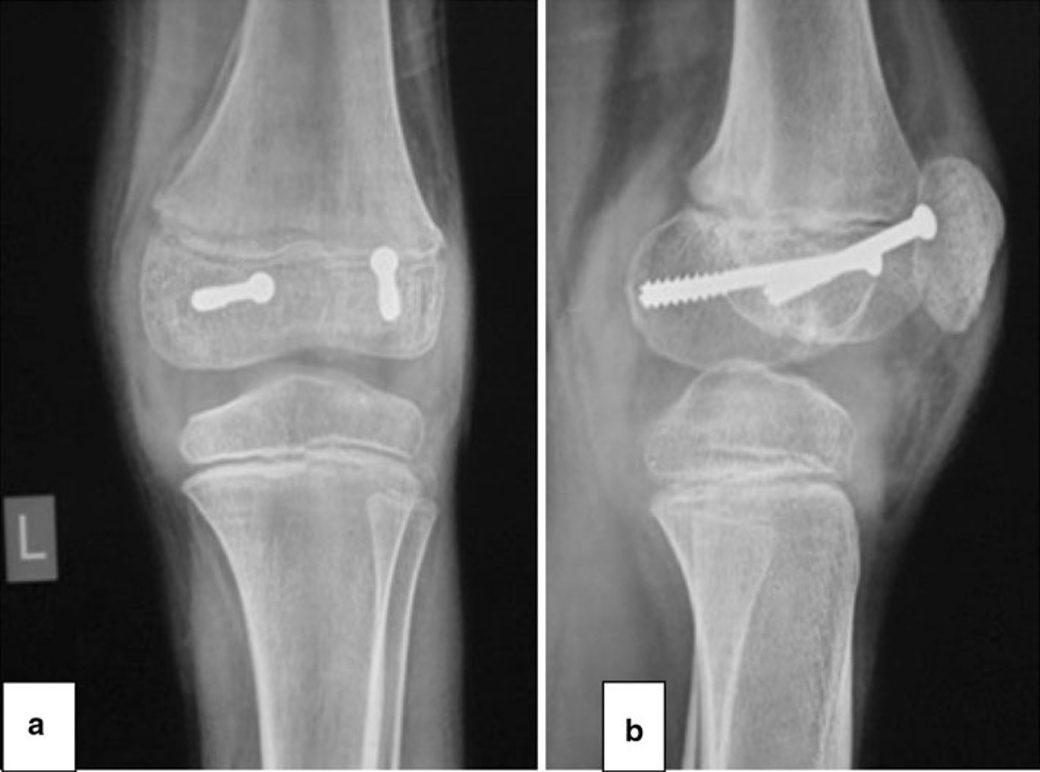
Anteroposterior **a** and lateral **b** radiographs showing the united fracture

**Fig. 5:**
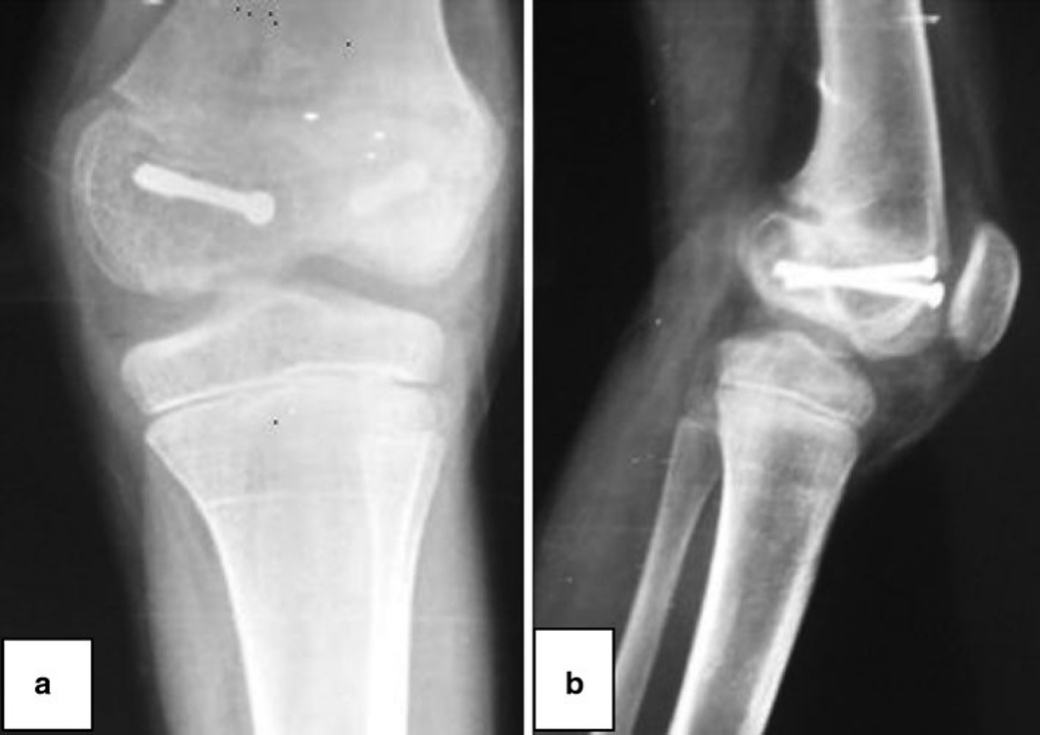
Final follow-up anteroposterior **a** and lateral **b** radiographs, showing anatomical union of the fracture site with no attendant osteoarthritic or avascular changes in the knee joint

Consent was obtained from the patient’s parents to publish his case, and the study was authorized by the local ethical committee and performed in accordance with the ethical standards of the 1964 Declaration of Helsinki as revised in 2000. A 9-year-old child presented to us with swelling of the left knee in the emergency department. The patient had a history of a fall from height onto a flexed knee. On physical examination, the knee was swollen with no external wound. There was tenderness over the lower end of the femur, and all movement at the knee joint was restricted and painful. There was no distal neurovascular deficit in the affected limb. Anteroposterior X-ray of the knee was apparently normal (Fig. 1a), but the lateral view revealed a horizontal coronal plane fracture of femoral condyle (Fig. 1b) of Letenneur type II. Arthroscopically assisted internal fixation of the lateral condyle was planned. To our surprise, during arthroscopy, we found a coronal fracture of both femoral condyles, and the two hemicondyles were conjoined by an intervening bone–articular cartilage bridge (Fig. 2). The bridge was formed from the part of the femur that participates in forming the distal aspect of the patellofemoral joint. Both the menisci and cruciate ligaments were normal. The fracture was reduced with the help of a hook (as used during routine arthroscopy), which was used to push the bridge between the two condyles suffering the Hoffa fracture, and the fracture was then held by percutaneous tenaculum clamps. After having achieved provisional reduction using two guide wires (Fig. 3), the fracture was fixed with 4.5-mm cannulated cancellous screws, inserted from anterior to posterior just distal to the femoral physis. Screws were placed under fluoroscopic control to prevent damage to the physis. The heads of the screws placed through the articular cartilage were countersunk. Postoperatively, a long plaster cast immobilization with the knee in 10° flexion was used for 2 weeks, followed by hinge brace application and progressive active physiotherapy of the knee. Weight bearing was allowed at 8 weeks, when the fracture had healed (Fig. 4a, b). At 3-year follow-up there were no signs of avascular necrosis of femoral condyle and the knee joint showed no radiological evidence of osteoarthritis (Fig. 5a, b).

## Discussion

Hoffa fractures are rare injuries, and lateral condyle fractures are more common than medial condyle fractures, probably because of physiological genu valgum; the lateral condyle is the most frequently injured condyle. Hoffa fracture can be associated with extensor mechanism injuries [5]. It usually occurs in adults and rarely affects children. The fracture results from a combination of forces: direct trauma, possibly with an element of abduction. Fall on a flexed knee concentrates the force in the posterior half of the femoral condyles, which is why this injury is more common in two-wheeled vehicle accidents where the knee is in a flexed and abducted position. Letenneur et al. [7] provided a classification for Hoffa fractures. Type I is a vertical fracture involving the entire condyle parallel to the posterior cortex of the femur. Type II is a fracture of variable size, horizontal to the base of the condyle. Type III is a fracture oblique to the femur. They reported the best results with internal fixation and the poorest results in type III. In the literature, only a single case of Hoffa fracture has been reported in children [8]. This is the first report of a conjoint Hoffa-type fracture involving both condyles in a child. Both plain radiographs and computed tomography may be useful in diagnosis and surgical treatment of these lesions. Routine anteroposterior radiographs usually miss the lesion, because the fracture may be obscured by the intact, anterior part of the condyle. Even lateral radiographs can miss the lesion, if the fracture is minimally displaced; moreover, a fracture of the type described herein may be misinterpreted as a unicondylar Hoffa fracture due to superimposition of the condyles.

It is generally accepted that surgical stabilization is necessary to achieve satisfactory function following Hoffa fracture, the reason being that reduction of the fracture fragment is difficult to achieve and maintain by closed reduction and casting/traction techniques, due to the absence of soft tissue attachment. For the same reason, this injury is also prone to avascular necrosis and nonunion, which are best prevented by stable anatomic compressive reduction and internal fixation, which is only possible by open/arthroscopic means. Operative treatment provides early functional rehabilitation and also decreases the chances of osteoarthritis, as evident from the final result in our case. For open reduction of bicondylar Hoffa fracture, the lateral approach is useful in most cases, but a standard anterior midline incision with medial parapatellar release and lateral dislocation of the patella, allowing direct access to the articular aspect of the fracture, also can be used. In the literature, there are reports of arthroscopically assisted reduction and internal fixation of Hoffa fracture [9]. Arthroscopic management reduces soft tissue dissection, blood loss, and operative time.

We conclude that conjoint-type bicondylar Hoffa fracture should be kept in mind in children also and must be managed operatively to prevent nonunion and deformity.
